# Determining the Catalytic Activity of Transition Metal-Doped TiO_2_ Nanoparticles Using Surface Spectroscopic Analysis

**DOI:** 10.1186/s11671-017-2355-7

**Published:** 2017-11-03

**Authors:** Sena Yang, Hangil Lee

**Affiliations:** 1 Center for Nano Characterization, Korea Research Institute of Standards and Science, Daejeon, 305-400 Republic of Korea; 2 Department of Chemistry, Sookmyung Women’s University, Seoul, 140-742 Republic of Korea

**Keywords:** Transition metal-doped TiO_2_, Catalytic activity, HRPES, STXM, EC measurements

## Abstract

The modified TiO_2_ nanoparticles (NPs) to enhance their catalytic activities by doping them with the five transition metals (Cr, Mn, Fe, Co, and Ni) have been investigated using various surface analysis techniques such as scanning electron microscopy (SEM), Raman spectroscopy, scanning transmission X-ray microscopy (STXM), and high-resolution photoemission spectroscopy (HRPES). To compare catalytic activities of these transition metal-doped TiO_2_ nanoparticles (TM-TiO_2_) with those of TiO_2_ NPs, we monitored their performances in the catalytic oxidation of 2-aminothiophenol (2-ATP) by using HRPES and on the oxidation of 2-ATP in aqueous solution by taking electrochemistry (EC) measurements. As a result, we clearly investigate that the increased defect structures induced by the doped transition metal are closely correlated with the enhancement of catalytic activities of TiO_2_ NPs and confirm that Fe- and Co-doped TiO_2_ NPs can act as efficient catalysts.

## Background

For several decades, it has been well known that titanium oxide (TiO_2_) has an effective catalytic activity as well as low cost, so TiO_2_ has received significant attention because of its various applications in solar cells, photocatalysis, and electrochemical catalysis [1–7]. Although TiO_2_ is a promising material, the TiO_2_ (rutile or anatase structures) has relatively wide band gap (E_g_ = 3.0~3.2 eV), and this width allows it to absorb only UV light. Therefore, significant efforts have been applied toward narrowing its band gap and enhancing catalytic activity. For this reason, an insertion of foreign elements as dopants has been widely performed to narrow the bandgap since the impurity element in TiO_2_ can modify band edge states.

Hence, our strategy is to insert transition metals as dopants into TiO_2_ NPs to enhance the catalytic performance of TiO_2_ NPs significantly, because they can increase the defect structures of TiO_2_ NPs, which is closely related to the enhancement of catalytic activity [8–18]. To further study from previous researches [19, 20], we performed the insertion of various transition metal ions (TM^+^) into TiO_2_ and then compared catalytic activities of the TiO_2_ NPs containing the various transition metal dopants with those TiO_2_ NPs. From this, we can assess the effectiveness of transition metal dopants for TiO_2_ NPs and compare photocatalytic activities between various transition metals together.

In our study, we successfully fabricated the five transition metal-doped TiO_2_ NPs (TM-TiO_2_; TM=Cr, Mn, Fe, Co, and Ni) with a thermo-synthesis process (see the “Methods” section). We first compared the morphologies and electronic properties of the five TM-TiO_2_ with TiO_2_ NPs by using scanning electron microscopy (SEM), Raman spectroscopy, and scanning transmission X-ray microscopy (STXM). And then, we assessed their catalytic capacities by oxidizing 2-aminothiophenol (2-ATP) under ultra-high vacuum (UHV) conditions (a base pressure below 9.5 × 10^−11^ Torr) with 365 nm UV light illumination using high-resolution photoemission spectroscopy (HRPES), and cyclic voltammogram (CV) changes in the solution phase by using electrochemistry. These reactions and analyses were also performed to determine the mechanism of the catalytic oxidation reaction.

## Methods

### Preparation of the Precursor Solutions

We prepared each precursor solution with a one pot synthesis. The desired amounts of the transition metal dopants (TM) were added in the form TM(NO_3_)_x_∙*n*H_2_O (metal nitrate *n*-hydrate; TM=Cr, Mn, Fe, Co, or Ni) as mole fractions with respect to TiO_2_ (TM/(TM+TiO_2_)), which were used as the dopants. All substances were purchased from Sigma-Aldrich. The precursor solutions are stirred for 10 min. 2-Aminothiophenol (2-ATP, Sigma Aldrich, 97% purity) and Nafion (Sigma Aldrich, 5 wt% in a low-molecular-weight aliphatic alcohol and water) were purchased from Sigma-Aldrich. Phosphate-buffered saline (PBS) tablets are purchased from Gibco.

### Preparation of the Dispersed TM-TiO_2_ Solutions

Tetramethylammonium hydroxide (TMAOH) (1.2 g) was diluted with double-distilled water (DDW, 22.25 g). Titanium isopropoxide (TTIP, 3.52 g) was diluted with isopropanol (3.5 g). Both of these solutions were stirred separately for 10 min. White TiO_2_ appeared by adding the TTIP solution dropwise to the TMAOH solution at room temperature. And then, the desired amounts (5 mol%) of the transition metal dopants were added to each synthetic gel solution in an oil bath at 80 °C with stirring. After approximately 10 min, the synthetic gel solution became a transparent solution. The solutions were transferred to Teflon-lined autoclaves and then heated at 220 °C for 7 h in a convection oven. The resulting TM-TiO_2_ (Cr-TiO_2_, Mn-TiO_2_, Fe-TiO_2_, Co-TiO_2_, and Ni-TiO_2_) were filtered and washed with DDW to remove any residue.

### Fabrication of TM-TiO_2_-Nafion-Modified GCE and Electrochemical Measurements of 2-ATP Oxidation

The electrochemical oxidation of 2-ATP was investigated using glassy carbon electrodes (GCEs) modified with TM-TiO_2_. For each TM, a mass of 4.0 mg of TM-TiO_2_ was dispersed into 2.0 ml of distilled water containing 50 μl Nafion, and then mixed by using an ultrasonic processor for 5 min to obtain the homogeneous TM-TiO_2_-Nafion mixture. After that, a volume of 20 μl of the mixture was placed on a GCE and was dried at 80 °C in a pre-heated oven for 30 min. A cyclic voltammogram (CV) of 0.01 M 2-ATP in PBS was obtained for each TM-TiO_2_-Nafion modified GCE.

### Characterizations

The morphology and size distribution of the fabricated nanoparticles was analyzed by using field-emission scanning electron microscopy (FE-SEM, FEI Inspect F50, operating at 10 kV). Raman spectra were obtained by using a spectrometer (Horiba, ARAMIS) with an Ar^+^ ion CW (514.5 nm) laser. Scanning transmission X-ray microscopy (STXM) results with a 25-nm resolution were obtained at the 10A beamline of the Pohang Accelerator Laboratory (PAL). STXM was used to obtain image stacks by using X-ray absorption spectroscopy (XAS) to elicit the doped transition metal *L*-edge, Ti *L*-edge, and O *K*-edge spectra. High-resolution photoemission spectroscopy (HRPES) experiments were carried out on an electron analyzer (SES-100, Gamma-Data Scienta) at the 8A2 beamline of PAL to identify the electronic structure. The S 2*p* core level spectra were recorded with an electron energy analyzer. A GCE with a diameter of 2 mm was used as the working electrode and a Pt wire with a diameter of 1 mm was used as the counter electrode, while the reference electrode was Ag/AgCl (3 M KCl).

## Results and Discussion

**Fig. 1:**
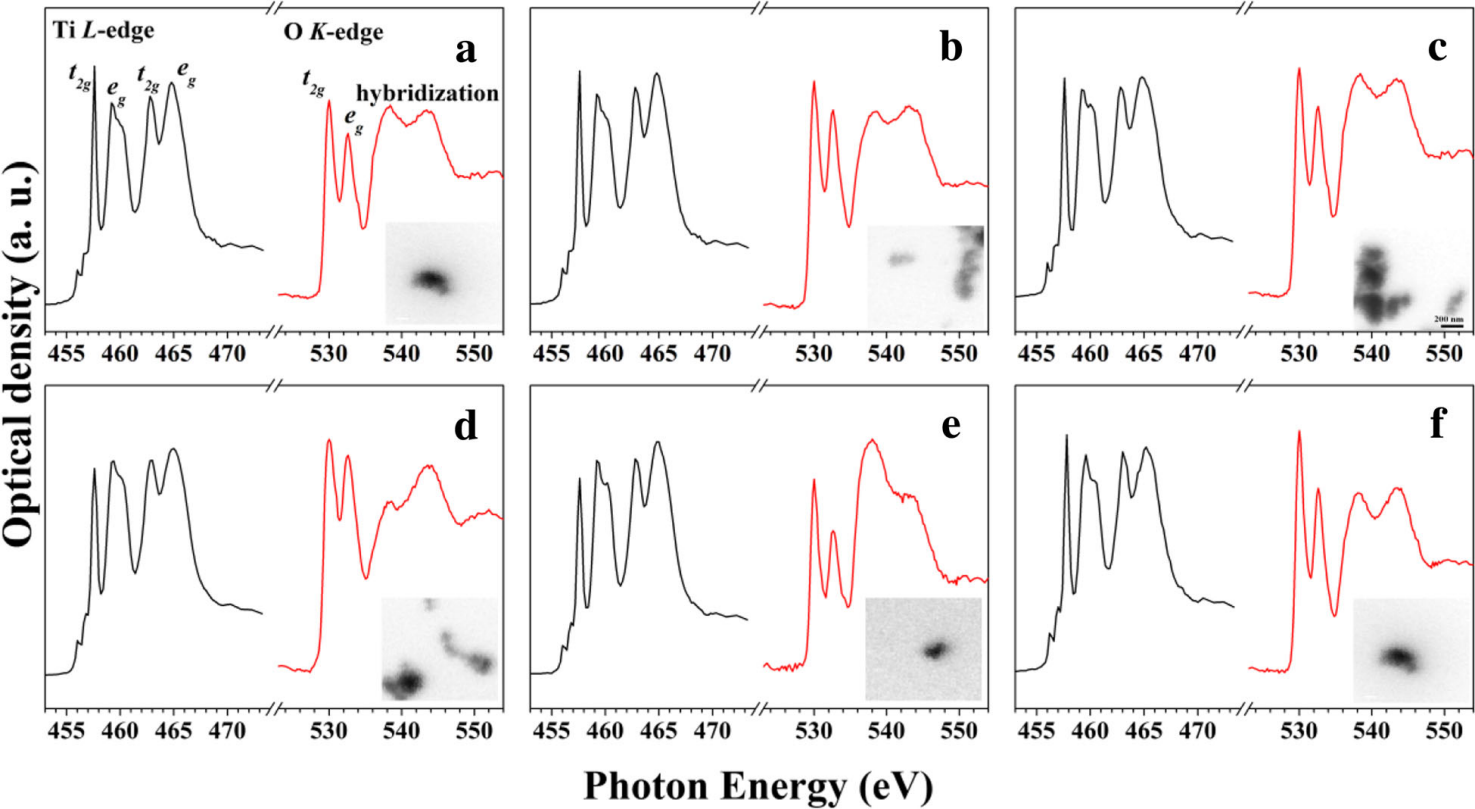
XAS spectra (Ti *L*_2,3_-edge and O *K*-edge) and the corresponding stacked images for **a** anatase TiO_2_, **b** Cr-TiO_2_, **c** Mn-TiO_2_, **d** Fe-TiO_2_, **e** Co-TiO_2_, and **f** Ni-TiO_2_ (5 mol% TM-TiO_2_ NPs). The stacked image size is 1 μm × 1 μm (scale bar is 200 nm)

To obtain more detailed characterizations of the electronic structures, we firstly obtained the Ti *L*-edge and O *K*-edge X-ray adsorption spectra (XAS) for TiO_2_ NPs and the five TM-TiO_2_ (Fig. 1) by using STXM. The black regions of the inset images shown in Fig. 1a–f are originated from TiO_2_ NPs and TM-TiO_2_. Firstly, the shape of the *e*_g_ orbital located at ~ 460 eV for the Ti *L*_2,3_-edge XAS spectra indicates the presence of typical anatase TiO_2_ structure in all TiO_2_ NPs and the five TM-TiO_2_ [21]. However, when TiO_2_ NPs are doped with Fe^3+^ (Fig. 1d) and Co^3+^ ion (Fig. 1e), the ratio of the intensities of the peaks *t*_2g_ (457.4 eV) and *e*_g_ (459~460 eV) decreases below those of the anatase TiO_2_ and other TM-TiO_2_ (Cr-TiO_2_, Mn- TiO_2_, and Ni-TiO_2_), which indicates the presence of a weak crystal field or an increment in the number of under-coordinated Ti atoms. In other words, these differences are due to the different dopants, which produce different defect structures in the nanoparticles. The small doublets at 456.0 and 456.6 eV in these figures correspond to the Ti^3+^ state; it is well known that metal doping enhances the surface defect structure [22, 23]. The O *K*-edge XAS spectra of the TiO_2_ NPs and five TM-TiO_2_ contain four peaks at 529.9, 532.3, 537.9, and 543.7 eV [24, 25]. As mentioned in the introduction, the principal purpose of this study is to investigate the electronic states of the TM-TiO_2_ and the effects on their catalytic activities. Interestingly, the O *K*-edge spectra show a quite different electronic structure depending on the transition metal dopants. As shown in O *K*-edges, peaks are due to the transition from the O 1*s* state to the unoccupied *p* state, and from the O 2*p* state to the O 2*p*–Ti 3*d* hybrid orbital state, respectively. The shapes and intensities of the O *K*-edge peaks for Cr-TiO_2_, Mn-TiO_2_, and Ni-TiO_2_ are very similar to those for anatase TiO_2_ NPs. However, the O *K*-edges of Fe-TiO_2_ and Co-TiO_2_ indicate less of the hybrid orbital (538 and 543 eV) than of the bare O 2*p* transition (532.6 eV). In other words, the orbitals of Fe and Co dopants are less hybridized with the O 2*p* orbital including TiO_2_ according to the spectra, which is related to catalytic activities and will be discussed again.

**Fig. 2:**
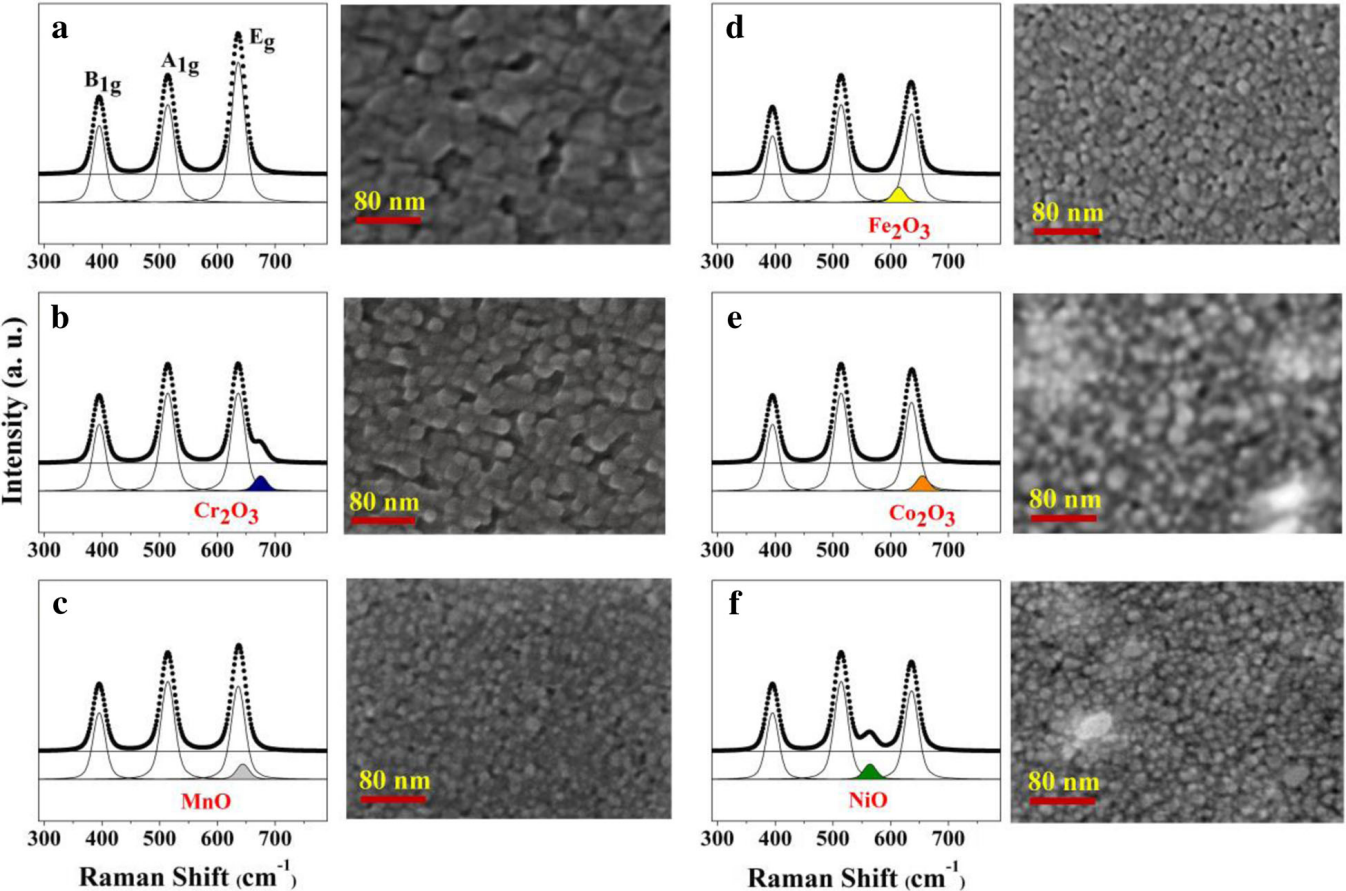
The Raman spectra of monodisperse 5 mol% TM-TiO_2_: **a** anatase TiO_2_, **b** Cr-TiO_2_, **c** Mn-TiO_2_, **d** Fe-TiO_2_, **e** Co-TiO_2_, and **f** Ni-TiO_2_ and the corresponding SEM images, respectively

We also measured the Raman spectra of TiO_2_ NPs and the five TM-TiO_2_. As shown in Fig. 2, the electronic structures among TM-TiO_2_ are also found to differ, compared with anatase TiO_2_ modestly, according to the Raman spectroscopic results. The six samples yield Raman shifts at about 395 (B_1g_), 514 (A_1g_), and 636 cm^−1^ (E_g_), and they indicate typical anatase TiO_2_ peaks [26]. Additionally, we found that each samples show doped transition metal-induced peaks (Cr_2_O_3_: 675.3 cm^−1^, MnO: 644.5 cm^−1^, Fe_2_O_3_: 614.2 cm^−1^, Co_2_O_3_: 657.1 cm^−1^, and NiO: 564.8 cm^−1^). Interestingly, we figured out that the doped transition metal ions were changed into the stable metal oxide forms, and the intensity of E_g_ peak of TiO_2_ NPs was a bit lower for TM-TiO_2_ than for anatase TiO_2_ NPs. We also acquired the SEM (Fig. 2) images of the TiO_2_ NPs and the five TM-TiO_2_ to determine their surface morphologies. The SEM images show that they have different structural features and sizes. Cr-TiO_2_, Mn-TiO_2_, Fe-TiO_2_, Co-TiO_2_, and Ni-TiO_2_ have uniform round or rectangular shapes with sizes of ~ 26, ~ 10, ~ 15, ~ 18, and ~ 16 nm, respectively. These five TM-TiO_2_ (TM=Cr, Mn, Fe, Co, and Ni) are significantly smaller than the anatase TiO_2_ NPs (~ 40 nm: Fig. 2a). Hence, it is possible that the Cr, Mn, Fe, Co, and Ni ions can modify the structure of the TiO_2_ NPs and then can act as nucleation sites that assist the formation of fine particles.

**Fig. 3:**
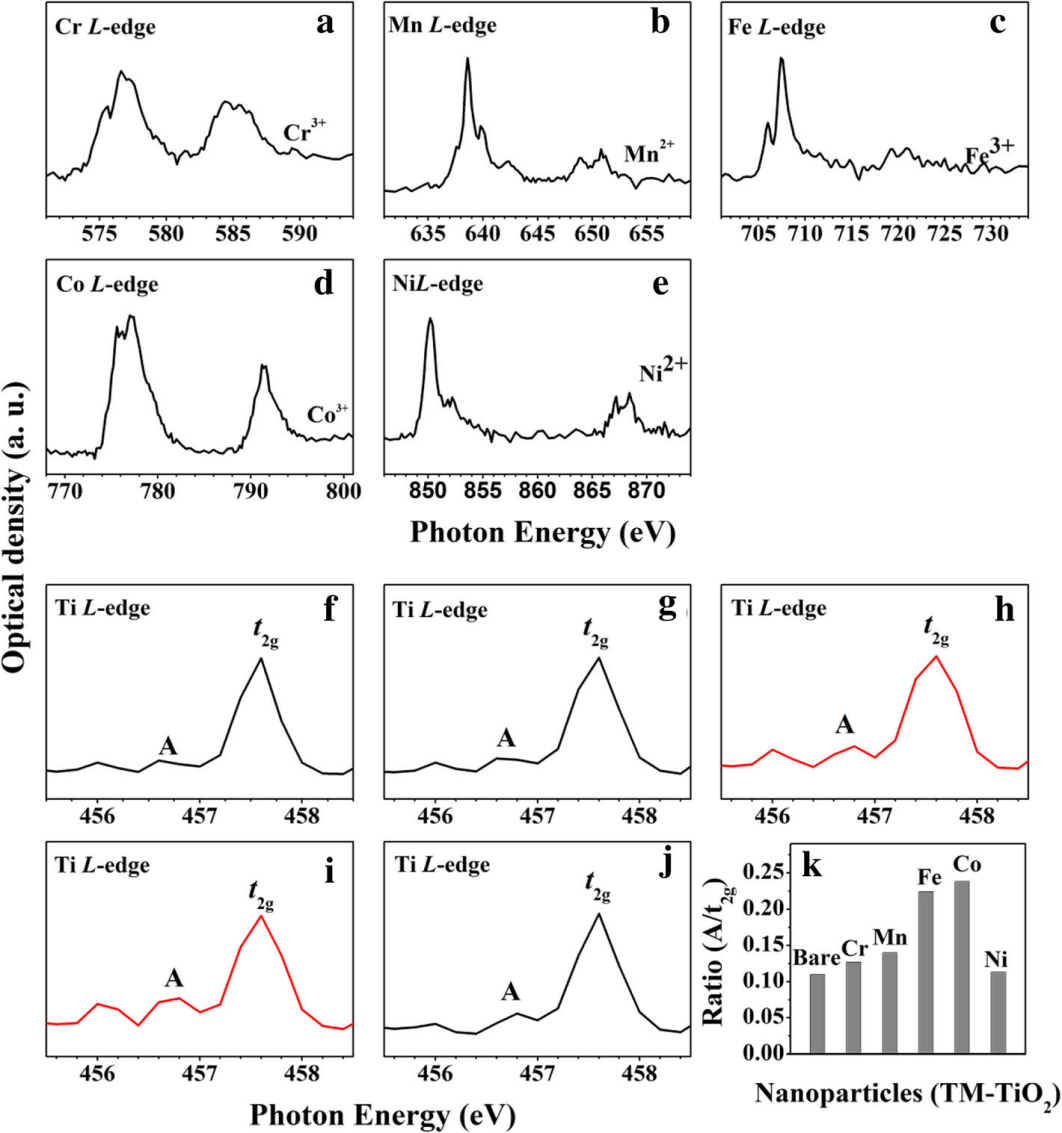
The doped transition metal *L*-edge and Ti *L*-edge XAS spectra of 5 mol% TM-TiO_2_: **a** and **f** Cr-TiO_2_, **b** and **g** Mn-TiO_2_, **c** and **h** Fe-TiO_2_, **d** and **i** Co-TiO_2_, and **e** and **j** Ni-TiO_2_. **k** The plot of ratio between pre-edge peak **a** and *t*_2g_ peak for bare TiO_2_ and the five TM-TiO_2_

In order to examine the modified electronic states induced by the transition metal dopants in more detail, we recorded the transition metal *L*-edge XAS spectra. Figure 3a–e clearly reveals the electronic structures of the five transition metal dopants being included in anatase TiO_2_ NPs. The spectrum in Fig. 3a with peaks at 576.0 and 577.0 eV with a 578.4-eV shoulder matches typical Cr^3+^ *L*_3_-edge results for Cr-TiO_2_ [27]. The sharp peak in Fig. 3b at 639.2 eV with a small feature at 640.7 eV matches other Mn^2+^ *L*_3_-edge results [28]. The sharp peak in Fig. 3c at 708.5 eV with a small peak at 706.6 eV matches other Fe^3+^ *L*_3_-edge results [29, 30]. The doublet in Fig. 3d at 776.8 and 777.6 eV is that of the Co^3+^ *L*_3_-edge [27]. Finally, the sharp peak at 850.3 eV in Fig. 3e with a small peak at 852.2 eV is the typical Ni^2+^ *L*_3_-edge spectrum [30]. These results establish the electronic states of the doped transition metals: Cr_2_O_3_, MnO, Fe_2_O_3_, Co_2_O_3_, and NiO, respectively.

One of our focus is to clarify the transition metal dopants induced defect structures of the TM-TiO_2_ in this study. As shown in Fig. 3f–j, we can notice that the intensities for the Fe-TiO_2_ and Co-TiO_2_ of the two pre-edge peaks at 456.7 and 457.4 eV are higher than those of Cr-TiO_2_, Mn-TiO_2_, and Ni-TiO_2_ (marked a) indicating that these peaks are due to surface defect structures (Ti^3+^ state) [31]. The ratios of the intensities of the pre-edge peak (a) and the *t*_2g_ peak are 0.11, 0.127, 0.140, 0.224, 0.238, and 0.113 for TiO_2_, Cr-TiO_2_, Mn-TiO_2_, Fe-TiO_2_, Co-TiO_2_, and Ni-TiO_2_, respectively (see Fig. 3k). This result means that the Ti^3+^ state is present in higher numbers in Fe-TiO_2_ and Co-TiO_2_.

**Fig. 4:**
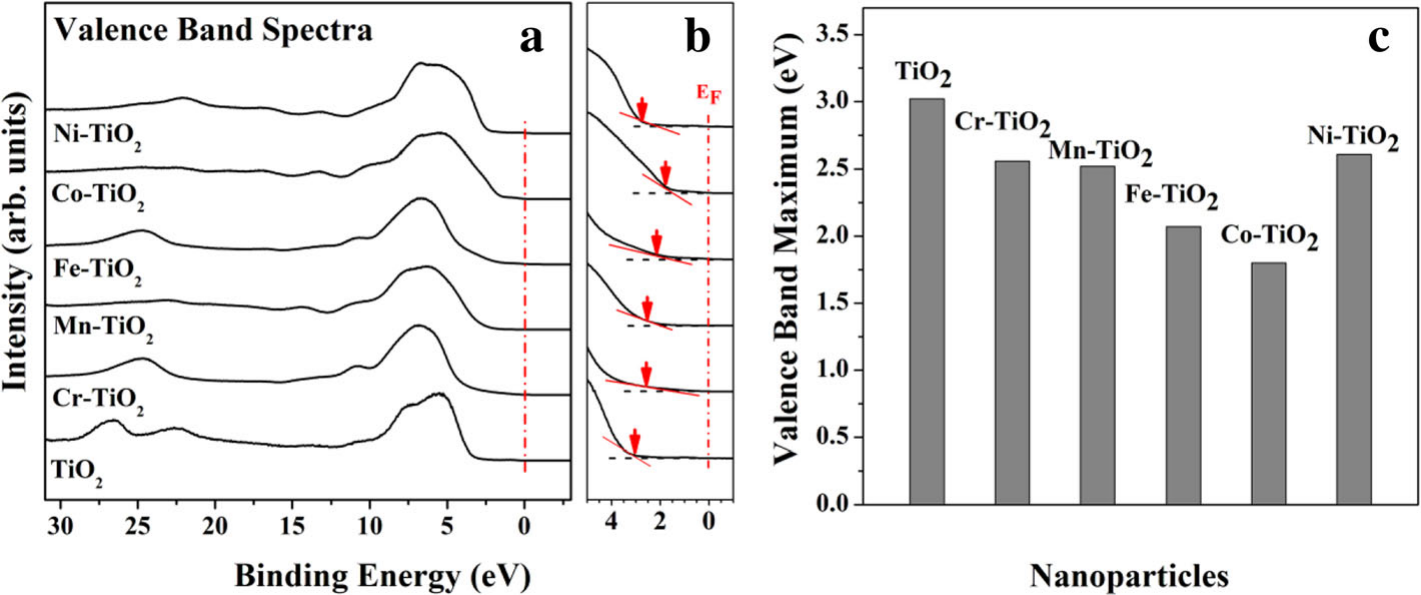
**a** Valence spectra and **b** magnified view of valence band edge of anatase TiO_2_ and the five TM-TiO_2_. **c** The plot of valence band maximum values of TiO_2_ and the five TM-TiO_2_

Following the confirmation of transition metal doping by the surface analysis, we investigated band gap modulations by taking the valence-band spectra as shown in Fig. 4. The anatase TiO_2_ has been reported to have a band gap of ~ 3.2 eV [32]. As shown in the valence-band spectra of Fig. 4a, the valence band maximum of TM-TiO_2_ shifts lower with respect to Fermi level (E_F_) from 3.10 to 1.81 eV (2.56 eV, Cr-TiO_2_; 2.52 eV, Mn-TiO_2_; 2.07 eV, Fe-TiO_2_; 1.81 eV, Co-TiO_2_; and 2.61 eV, Ni-TiO_2_). From this, we can estimate that the transition metal doping gives rise to band gap narrowing because TiO_2_ is highly n-type semiconductor material, and E_F_ in the n-type semiconductor lies close to the conduction band. Narrowing the band gap of TM-TiO_2_ has resulted from its enhancement of defect structures.

As a result, we can conclude that the doped transition metals make defect structures of TiO_2_ NPs and then contribute to decrease the band gap in TM-TiO_2_ (in special Fe-TiO_2_ and Co-TiO_2_). With these understanding of variations of the structures and electronic properties for the five TM-TiO_2_, we now compare the effects of transition metal doping as a point of their catalytic activities.

### Electrochemical Redox Reaction in the Aqueous Phase

**Fig. 5:**
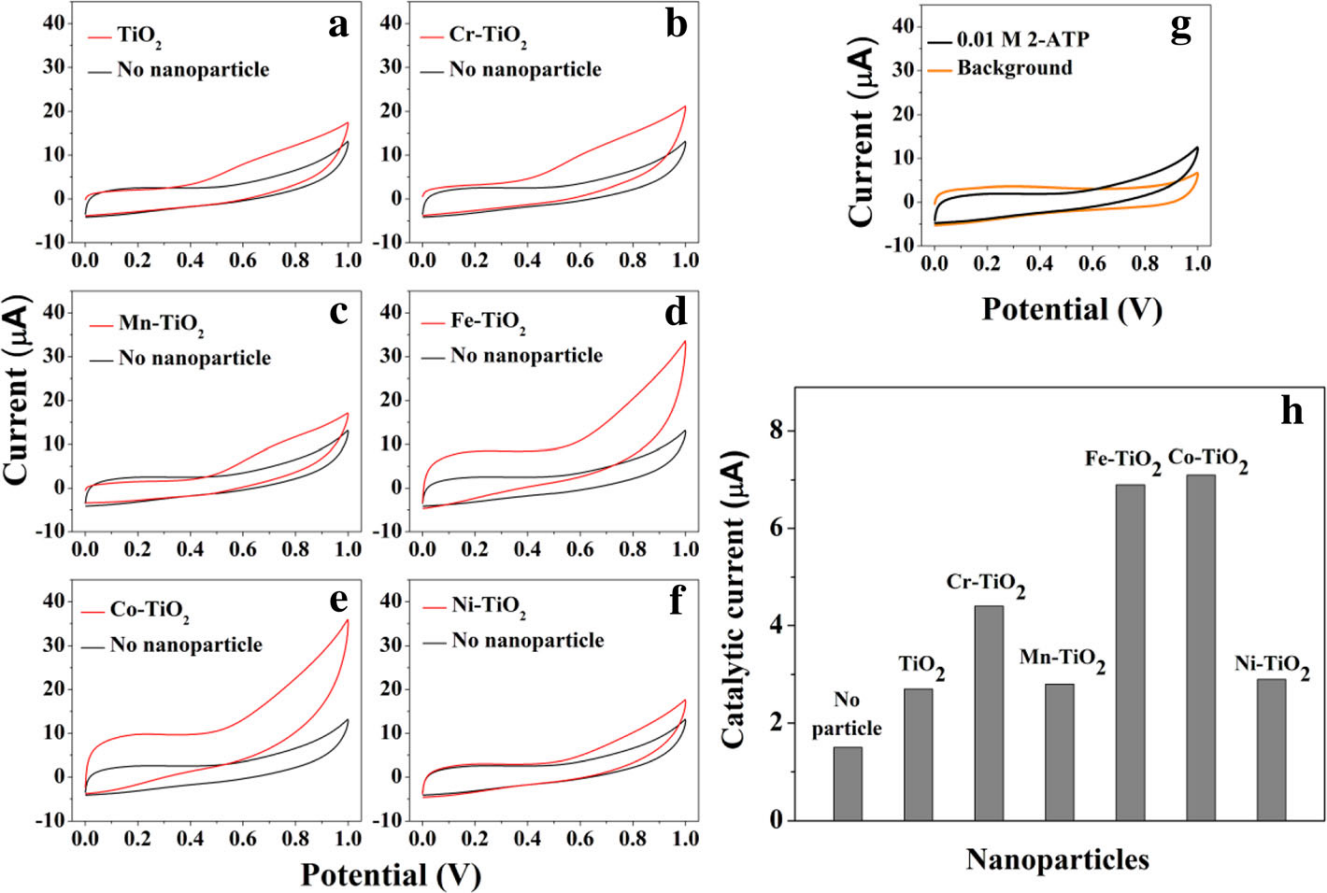
**a**–**f** CVs (at a scan rate of 50 mV/s) in PBS containing 0.01 M 2-ATP at a bare GCE (black lines) or GCEs modified (red lines) with 5 mol% **a** anatase TiO_2_, **b** Cr-TiO_2_, **c** Mn-TiO_2_, **d** Fe-TiO_2_, **e** Co-TiO_2_, and **f** Ni-TiO_2_. **g** A sluggish oxidation current observed at a bare GCE because of the intrinsically slow oxidation of 2-ATP. **h** Catalytic currents resulting from the electrochemical oxidation of 2-ATP for the various types of anatase TiO_2_ and the five TM-TiO_2_

CVs were obtained in a PBS solution containing 0.01 M 2-ATP at various types of GCEs irradiated by 365-nm-wavelength UV light. As shown in Fig. 5g, a sluggish oxidation current is observed at a bare GCE because of the intrinsically slow oxidation of 2-ATP. To increase the current associated with the oxidation of 2-ATP, GCEs modified with the TiO_2_ and TM-TiO_2_-Nafion catalysts are fabricated and tested, with the results shown in Fig. 5. The currents associated with the oxidation of 2-ATP are 6.9 (± 1.4) μA and 7.1 (± 1.6) μA when using the GCEs modified with the Fe-TiO_2_ and Co-TiO_2_, respectively—significantly greater (i.e., 4.6 and 4.7 times greater) than the 2.0 μA value observed when using only the bare GCE (Fig. 5h). In contrast, the currents generated when using the anatase TiO_2_ NPs, Cr-TiO_2_, Mn-TiO_2_, and Ni-TiO_2_ are only 2.7 (± 0.4) μA, 4.4 (± 1.1) μA, 2.8 (± 0.5) μA, and 2.9 (± 0.7) μA, respectively, which are slightly (1.8, 2.9, 1.86, and 1.93 times) but not significantly greater than that for the bare electrode. These results reveal the importance of the type of TM-TiO_2_ for catalyzing oxidation reactions, even when using small amounts (5 mol%) of the doped transition metal, and specifically indicate the Fe-TiO_2_ and Co-TiO_2_ to be good catalysts for the oxidation of 2-ATP.

### Photocatalytic Oxidation of 2-ATP

**Fig. 6:**
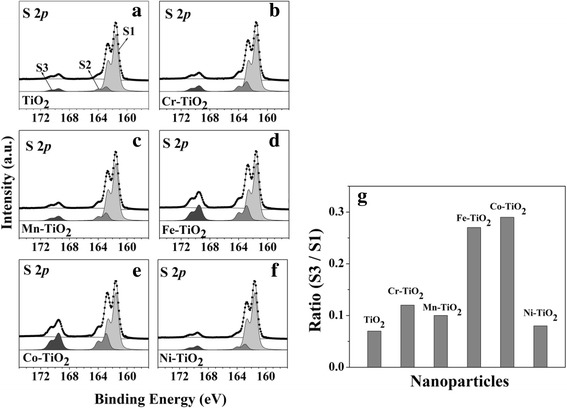
(Left panel) HRPES S 2*p* core-level spectra obtained after the catalytic oxidations of 180 L 2-ATP (the saturation exposure in our system) on anatase TiO_2_ and 5 mol% TM-TiO_2_ (**a** TiO_2_, **b** Cr-TiO_2_, **c** Mn-TiO_2_, **d** Fe-TiO_2_, **e** Co-TiO_2_, and **f** Ni-TiO_2_). (Right panel) **g** The plot for intensity ratio between S3 (− SO_3_H) and S1 (− SH) of anatase TiO_2_ and the five TM-TiO_2_, indicating their catalytic activities in the oxidation of 2-ATP, for 180-l exposures under 365 nm UV light

We also determined the direct catalytic activities of the TM-TiO_2_ in the oxidation of 2-ATP molecules. The S 2*p* core-level spectra of anatase TiO_2_ and 5 mol% TM-TiO_2_ were obtained with HRPES after 180 l of 2-ATP exposure in the presence of oxygen under 365 nm UV light illumination (see Fig. 6a–f). These spectra contain three distinct 2*p*_3/2_ peaks at 161.5, 162.9, and 168.6 eV, which are assigned to S1, the C-SH unbounded state, S2, the bound state, and S3, sulfonic acid (SO_3_H), respectively. It is well known that sulfonic acid is an oxidation product of thiol groups [33, 34]. Hence, we can monitor the oxidation of 2-ATP by measuring the ratio of the intensities of peaks S3 and S1. Figure 6a–f confirms that Fe-TiO_2_ and Co-TiO_2_ act as effective photocatalysts. The ratios of the intensities are 0.07, 0.12, 0.10, 0.27, 0.29, and 0.08 for anatase TiO_2_ NPs, Cr-TiO_2_, Mn-TiO_2_, Fe-TiO_2_, Co-TiO_2_, and Ni-TiO_2_, respectively, i.e., the ratios of Fe-TiO_2_ and Co-TiO_2_ are also higher than those of the other nanoparticles (see Fig. 6g). This result is closely correlated with the number of defect structures in the TM-TiO_2_ shown in Fig. 3. In the STXM measurements, we confirm that Fe-TiO_2_ and Co-TiO_2_ contain more Ti^3+^ defect states (i.e., surface defect structures). Thus, these results indicate that increasing the number of Ti^3+^ defect structures is closely correlated with the enhancement of catalytic activity [7]. As a result, the Fe-TiO_2_ and Co-TiO_2_, which contain many Ti^3+^ defect states, have higher catalytic activities.

For this reason, we can consider three factors (charge state dependence, surface defect structure dependence, and hybridization between the doped transition metals and TiO_2_), which can cause the enhancement of catalytic activities of TM-TiO_2_. At first, the effect of electronic charge state has been also investigated by using STXM measurement. As shown in Fig. 3a–e, we confirm that Cr, Fe, and Co transition metal ions have the TM^3+^ charge states, while Mn and Ni have the TM^2+^ charge states. Therefore, we can conclude that there is no correlation between electron charge states of dopants and catalytic activity of TM-TiO_2_. Secondly, we checked the surface defect structure dependence. Comparing the ratio of the intensities of the pre-edge peak (A) and the *t*_2g_ peak shown in Fig. 3, we confirm that the number of surface defect structure is in order of Co-TiO_2_ > Fe-TiO_2_ > Mn-TiO_2_ > Cr-TiO_2_ > Ni-TiO_2_ > TiO_2_. As previously stated, Fe-TiO_2_ and Co-TiO_2_ exhibit clear enhancement in catalytic activity. With increasing surface defect structures, the catalytic activities of TM-TiO_2_ increase. By monitoring the pre-edge ratios, we observed clear surface defect structure dependence in enhancing catalytic activity. Consequentially, the surface defect structure only influences on enhancement of catalytic activity of TM-TiO_2_.

Finally, another reasonable explanation is that according to the O *K*-edge XAS shown in Fig. 2, a higher proportion of less-hybridized oxygen states (538 and 543 eV) appears in Fe-TiO_2_ and Co-TiO_2_ than in the other TM-TiO_2_. Those transition of the doped transition metal 3*d* to the O 2*p* unoccupied state can facilitate the removal of oxygen atoms from the TiO_2_ nanoparticles and enhance the catalytic oxidation of 2-ATP because oxygen vacancy site of TiO_2_ is an active site. Conclusively, doping the TiO_2_ nanoparticle with either Fe or Co yields a higher increase in the catalytic activities for 2-ATP oxidation than doping with Cr, Mn, or Ni.

## Conclusions

TM-TiO_2_ synthesized with a thermo-synthesis method were examined with various surface analysis techniques. To compare the catalytic activities of the five TM-TiO_2_ with the anatase TiO_2_ NPs, we monitored their effects on the photocatalytic oxidation of 2-ATP molecules by using HRPES and oxidation of 2-ATP by using EC measurements. Depending on the doped transition metals, we clearly investigated that the increased defect structures and less hybridization induced by the doped transition metals affect the enhanced catalytic activities. In particular, Fe^3+^ and Co^3+^ ions generate more effective oxidation state discrepancies, i.e., more Ti^3+^ defect structures and surface transformations than the other metal ions (Cr^3+^, Mn^2+^, and Ni^2+^). As a result, we figured out that the catalytic properties of Fe-TiO_2_ and Co-TiO_2_ are superior to those of anatase TiO_2_ NPs and other TM-TiO_2_ (TM=Cr, Mn, and Ni).

## Abbreviations

HRPES: High-resolution photoemission spectroscopy
SEM: Scanning electron microscopy
